# Muscle activity of deep hip muscles across joint positions and elevation phases during side-lying hip abduction

**DOI:** 10.7717/peerj.21536

**Published:** 2026-07-07

**Authors:** Kazuma Uebayashi, Yu Okubo, Kiyokazu Akasaka, Gen Adachi, Tomoki Oshikawa, Naoto Matsunaga, Koji Kaneoka

**Affiliations:** 1 Graduate School of Medicine, Saitama Medical University, Moroyama, Saitama, Japan; 2 Department of Rehabilitation, Suzuki Clinic Orthopedics Rivercity, Chuo, Tokyo, Japan; 3 Faculty of Health and Sports Sciences, Toyo University, Kita, Tokyo, Japan; 4 Center of General Education, Tokyo Keizai University, Kokubunji, Tokyo, Japan; 5 General Education Core Curriculum Division, Seigakuin University, Ageo, Saitama, Japan; 6 Faculty of Sports Science, Waseda University, Tokorozawa, Saitama, Japan

**Keywords:** Fine-wire EMG, Hip abductor, Muscle function, Exercise, Rehabilitation

## Abstract

**Introduction:**

Hip abductor dysfunction is associated with various musculoskeletal disorders, such as knee overuse injury, patellofemoral pain syndrome, and hip osteoarthritis. Thus, hip abduction exercise is used to improve hip muscle performance. Notably, deep hip muscles might be effectively activated during the hip internal rotation (IR) position. Moreover, it is hypothesized that hip abduction combined with hip IR might activate the deep hip muscles and minimize tensor fascia latae (TFL) activation.

**Objective:**

This study aimed to investigate the percentage maximum voluntary isometric contraction (%MVIC) and the activity ratio of the gluteus muscles in relation to the activity of the TFL—including the gluteus minimus (GMin), piriformis, and gluteus medius (GMed)—across different hip positions during side-lying hip abduction.

**Methods:**

Twelve healthy men performed hip abduction exercises in nine hip joint positions. The muscle activities of the lower extremities, including the anterior and posterior segments of the GMin (GMin-a and GMin-p) and piriformis, GMed, and TFL, were recorded *via* surface and fine-wire electromyography.

**Results:**

The %MVIC of the GMin-p and GMed was highest in hip extension and IR (EIR) position during the late concentric phase (GMin-p 54.7 ± 27.2 %MVIC; GMed 59.2 ± 36.1 %MVIC; *P* < 0.05). No significant difference was observed in the %MVIC of TFL (lower in the hip internal rotation position). The relative activation ratios (RARs) of the GMin-p and GMed to TFL were highest in hip position involving IR during the early concentric or late eccentric phases (GMin-p 3.80 ± 3.65; GMed 2.62 ± 1.72; <0.05).

**Conclusions:**

Hip abduction during the late concentric phase with EIR position caused maximal hip abductor activity. Conversely, hip abduction during the early concentric phase involving the IR position selectively activated the GMin-p and GMed while minimizing TFL activation.

## Introduction

Hip abductor dysfunction is associated with various musculoskeletal disorders, including knee overuse injury, patellofemoral pain syndrome, groin injury, low back pain, and hip osteoarthritis (Cooper et al., 2016; Kollock et al., 2016; Marshall et al., 2016; Selkowitz, Beneck & Powers, 2022, 2023; Whittaker et al., 2015). Specifically, the gluteus minimus (GMin) and piriformis (PIRI) are essential in hip abductor movement. A study investigating the activity pattern of the GMin in gait, by classifying the GMin into two segments (anterior [GMin-a] and posterior [GMin-p]), demonstrated that the contribution of the femoral head-stabilizing role was attributable to GMin-p activation in the early stance and co-contraction of GMin-a and GMin-p in the late mid-stance (Semciw et al., 2014). Other studies have suggested that because the GMin contributes to hip joint stability during gait, changes in GMin activity pattern may lead to muscle and joint injuries during functional tasks requiring larger strides, such as running (Semciw, Green & Pizzari, 2015). Furthermore, another study reported that the primary function of the GMin and PIRI muscles was the stabilization of the femoral head in the acetabulum at different hip positions during the gait cycle (Gottschalk, Kourosh & Leveau, 1989; Snijders, Hermans & Kleinrensink, 2006). Anatomical studies have suggested that the GMin can be regarded as a dynamic stabilizer *via* the capsular ligament (Tsutsumi, Nimura & Akita, 2020). Moreover, the structural features of PIRI reportedly support the role of the hip stabilizer (Parvaresh et al., 2019). Thus, these muscles are not only involved in hip abduction but also play a significant role in hip joint stabilization. Therefore, exercises that focus on the GMin and PIRI are essential in rehabilitation and training.

Gluteal muscle exercises are a fundamental component of rehabilitation for various lower limb musculoskeletal conditions. Gluteal muscle activity has been extensively investigated across a wide range of therapeutic exercises, including step-ups, lunges, squats, and clamshells (Distefano et al., 2009; French, Dunleavy & Cusack, 2010; Reiman, Bolgla & Loundon, 2012; Stastny et al., 2015, 2016; Ebert et al., 2017; Macadam & Feser, 2019). Stastny et al. (2016) highlighted that exercise selection should be guided not only by the magnitude of gluteal activation but also by the activity ratio between the tensor fascia latae (TFL) and gluteal muscles. Recently, excessive contraction of the TFL has been associated with musculoskeletal disorders such as patellofemoral pain syndrome (Selkowitz, Beneck & Powers, 2013, 2022, 2023). Furthermore, hip abduction exercises that activate the gluteus muscles while minimizing TFL activity have been recommended (Selkowitz, Beneck & Powers, 2013). A previous study on hip abduction exercises reported that clamshell exercise activates the gluteus maximus (GMax) and gluteus medius (GMed) while minimizing TFL activity (Selkowitz, Beneck & Powers, 2013, 2023). In addition, some studies have reported that hip abduction in the side-lying position promotes higher muscle activity in the GMin, especially in the posterior segment, than clamshell exercises (Moore et al., 2019; Semciw et al., 2014). Furthermore, isometric hip abduction exercise in double-leg standing sufficiently activates GMin to improve muscle strength (Ganderton et al., 2017). A recent study using musculoskeletal modeling further demonstrated that while weight-bearing exercises such as single-leg squats impose high peak forces, the side-lying leg raise is an exercise for specifically recruiting the gluteus minimus and medius (Colliings et al., 2023). Therefore, hip abduction exercises in the side-lying position may be effective in activating the deep hip muscles. However, hip abductors change the moment arm of a joint or muscle length by altering the hip joint position (Beck et al., 2000; Delp et al., 1999; Neumann, 2010). Specifically, the GMin and PIRI increase the moment arm of internal rotation (IR) with hip flexion (Delp et al., 1999; Neumann, 2010). In addition, during gait on the stance side, the GMin produces an IR torque, allowing for combined hip abduction and IR torque (Neumann, 2010). Thus, it is hypothesized that hip abduction combined with hip IR might activate the deep hip muscles and minimize TFL activation. This study aimed to investigate the muscle activities of hip abductors—including GMin and PIRI muscles—and to determine the relative activation ratio (RAR) for each gluteal muscle-to-TFL in various hip positions.

## Materials and Methods

### Study design

This cross-sectional study aimed to elucidate the muscle activities of hip abductors and the RAR for each gluteal muscle-to-TFL in nine hip positions. We used two independent variables (joint position and phase) and two dependent variables (muscle activity and RAR) for the analysis.

### Participants

A power analysis by G*power 3.1.9.6 (Heinrich Heine Universität Düsseldorf, Düsseldorf, Germany) was used to determine the sample size for the two-way repeated measures analysis of variance (ANOVA). The sample size was estimated at 12 people to be conducted *via* the two-way repeated ANOVA (three positions × five phases: effect size f of 0.40, α level of 0.05, power level of 0.8). Considering the potential challenges in analysis due to the use of fin-wire electrodes, we included 12 healthy men (mean ± standard deviation; age: 24.4 ± 2.8 years; height: 172.6 ± 5.0 cm; body mass: 64.8 ± 7.5 kg; body mass index, 21.7 ± 2.1 kg/m^2^). Healthy individuals without a history of lower back pain, lower extremity surgery or injury, spinal surgery, or neurological disorders were included. All participants provided written informed consent after receiving a detailed explanation of the purpose, potential benefits, and risks of participation. This study was conducted in accordance with the tenets of the Declaration of Helsinki and approved by the Ethics Review Committee on Research with Human Subjects of Waseda University (approval number: 2017-164).

### Electromyography (EMG)

**Figure 1:**
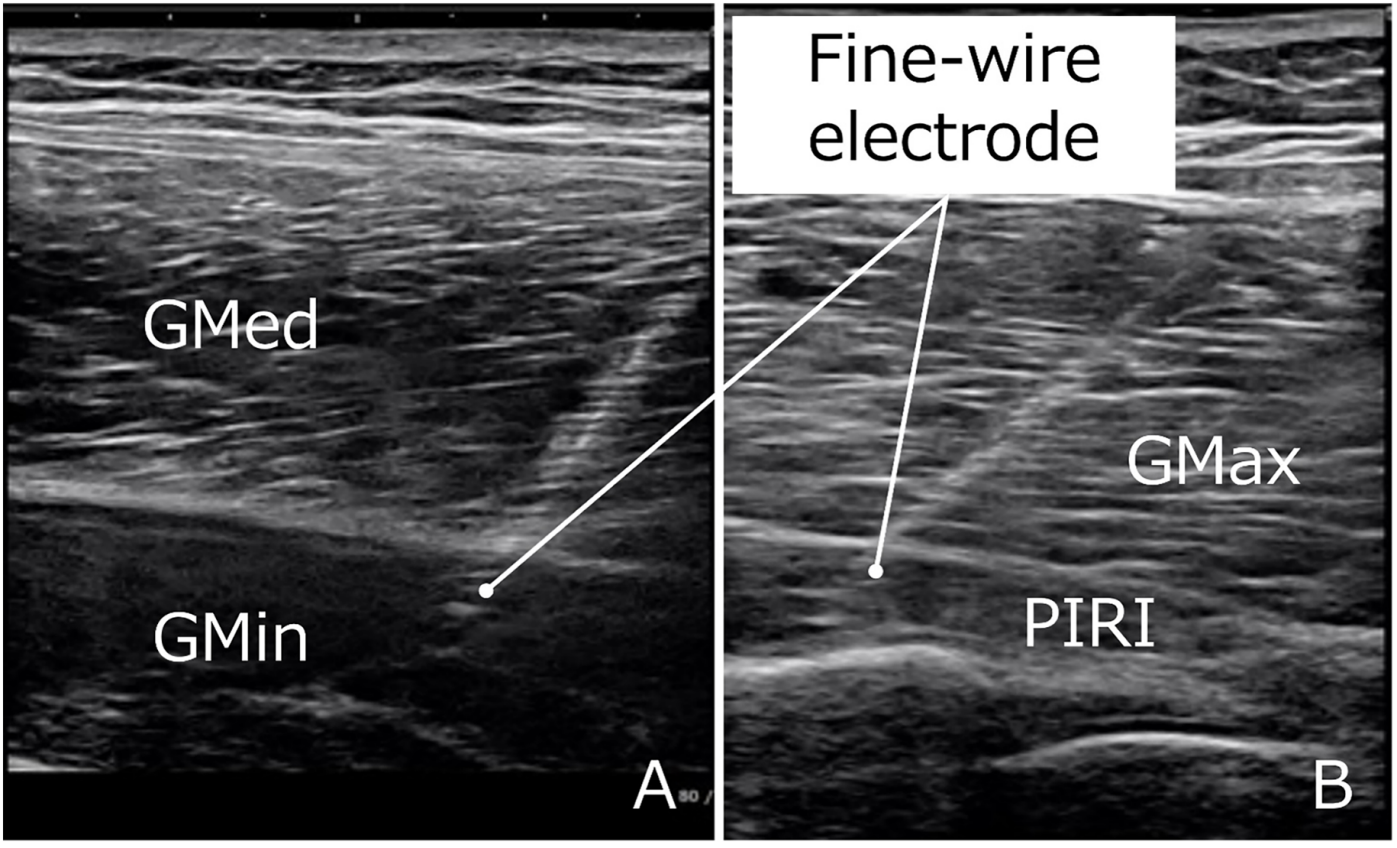
Ultrasound imaging showing insertion of fine-wire electrodes into the (A) GMin and (B) PIRI. GMin, gluteus minimus; PIRI, piriformis.

The electromyographic activities of the right GMin-a, GMin-p, and PIRI were recorded using intramuscular fine-wire electrodes, while those of the right TFL, GMax, GMed, and rectus femoris (RF) were recorded using a surface electrode. Bipolar electrodes were produced using two strands of urethane-coated stainless-steel wire with a 0.05-mm diameter (Unique Medical Co., Ltd., Tokyo, Japan). These electrodes were threaded into a hypodermic needle (23 gauge × 60 mm). Afterward, 1 mm of urethane was removed, and the tips of the fine wires were bent backward to create 1- and 2-mm hooks. Fine wires and needles were sterilized at 121 °C for 20 min in an autoclave. For electrode insertion, the participants were placed in a side-lying position on a bed with the hips and knees at 45° flexion and a pillow placed between the knees. An orthopedic doctor inserted the sterilized fine-wire electrodes into the target muscles *via* ultrasound imaging (SONIMAGE HS1 PRO, KONICA MINOLTA Co., Ltd., Tokyo, Japan) as follows: GMin-a, dividing the line connecting the anterior superior iliac spine (ASIS) and posterior superior iliac spine (PSIS) into six parts, and 3 cm inferiorly toward the greater trochanter (GT) from the one-sixth of the ASIS side of the direct line connecting the ASIS and PSIS (Fig. 1A) (Semciw et al., 2012); GMin-p, 3 cm inferiorly towards the GT from the midpoint of the direct line connecting the ASIS and PSIS (Fig. 1A) (Semciw et al., 2012); and PIRI, midpoint of the direct line between the posterior inferior iliac spine and posterior superior margin of the GT (Fig. 1B) (Giphart et al., 2012).

Pairs of disposable Ag/AgCl surface electrodes (BlueSensor N-00-S, Mets Co., Ltd., Tokyo, Japan) were attached 20 mm apart to the skin, parallel to the direction of the muscle fibers. Placement of the surface electrodes of the GMax, GMed, TFL, and RF was performed according to the methods of Surface Electromyography for the Non-Invasive Assessment of Muscles (Hermens et al., 2000). The wireless EMG telemeter system (BioLog DL-5000, S&ME Co., Tokyo, Japan), with a 2,000-Hz sampling rate, was used to measure fine-wire and surface EMGs. The characteristics of the system include: drop off at the ends of the bandwidth boundaries; 5–1,000 Hz, gain; 240 times, common mode rejection ratio; 80 dB, input impedance; 200 MΩ. Raw data were band-pass filtered (20‒950 Hz) and full-wave rectified using an EMG analysis software (BIMUTAS-Video; KISSEI COMTEC Co., Ltd., Nagano, Japan). The filtering bandwidth and sampling rate were selected in accordance with the Consensus for Experimental Design in Electromyography (CEDE) project recommendations to ensure the inclusion of the full power spectrum for both intramuscular and surface EMG signals while minimizing movement artifacts (Besomi et al., 2020).

### Kinematics

Kinematic data were collected to divide the task into phases and recorded using a three-dimensional motion analysis system (OQUS Motion System; QUALYSIS, Co., Ltd., Gothenburg, Sweden). Eight cameras were placed around each participant. In addition, two reflective markers were attached to the lateral epicondyle of the knee and the GT of the femur on the right side. The displacement of the femoral angle between the line connecting the markers on the lateral epicondyle and the GT and the horizontal plane was calculated to classify the phases of each hip abduction task. Based on the displacement of the femoral angle, hip abduction was classified into concentric and eccentric phases, and each phase was divided into two parts, with the first half being the early phase and the second half being the late phase. The sampling frequency was set to 200 Hz.

### Maximum voluntary isometric contraction (MVIC)

The participants performed maximum voluntary isometric contraction (MVIC) of each muscle for 3 s against maximum manual resistance. Muscle activity was observed and recorded while the examiner applied the resistance. MVIC tests for the GMin-a, GMin-p, and PIRI were performed using a method described in previous studies. For the GMin-a and GMin-p, participants in the side-lying position and maintaining a straight leg (hip flexion/extension 0°, rotation 0°, abduction 30°) performed maximum hip abduction (Semciw et al., 2014). Both GMin-a and GMin-p were normalized using the same MVIC task to ensure a valid comparison of activation levels between the two segments. For the PIRI, participants in the side-lying position and maintaining 90° knee flexion and 45° hip flexion performed maximum hip external rotation (ER) with both heels together (Morimoto et al., 2018). Each MVIC test was randomly performed once, with a resting period between each test. The resting period between MVIC tests was set to >1 min to account for fatigue. Raw waveforms were monitored during MVIC testing. Therefore, if noise occurred during the test, it was marked as a failed attempt and retested. Amplitude normalization was performed using the “MVIC-based normalization” approach, following the normalization matrix recommended by the CEDE project (Besomi et al., 2020). For each muscle, the root mean square (RMS) amplitude during the tasks was expressed as a percentage of the peak 1 s RMS obtained during the 3 s MVIC trial (%MVIC).

### Experimental task

**Figure 2:**
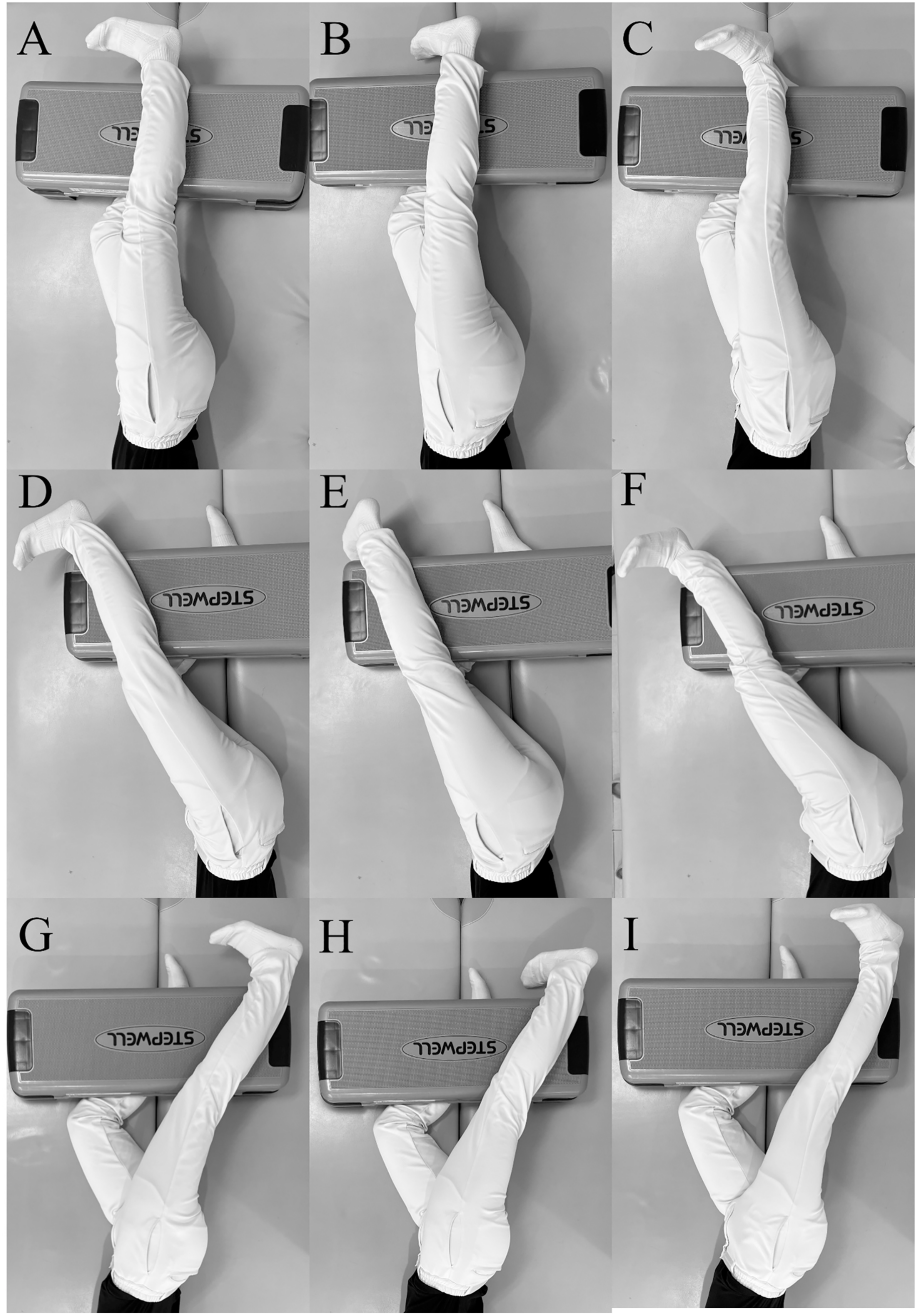
Hip abduction tasks in the side-lying position across nine hip positions. (A) Hip neutral position: 0° flexion/extension; (B) hip neutral position: 0° flexion/extension and maximum external rotation position; (C) hip neutral position: 0° flexion/extension and maximum internal rotation position; (D) hip 30° flexion and neutral position: 0° rotation; (E) hip 30° flexion and maximum external rotation position; (F) hip 30° flexion and maximum internal rotation position; (G) hip 15° extension and neutral position: 0° rotation; (H) hip 15° extension and maximum external rotation position; and (I) hip 15° extension and maximum internal rotation position.

All participants in the left-sided lying position randomly performed hip abduction in nine positions (Fig. 2). The starting position involved placing the elevated side on a step stand, setting the hip joint at 0°, and placing the contralateral side in a slightly flexed position. The hip joint angles for each task were standardized according to the neutral-zero method and verified using a goniometer by a physiotherapist. Hip abduction (30°) was performed without knee bending for 3 s (concentric phase). Participants kept their legs elevated for 3 s (hold phase) and placed them down afterward for 3 s, without bending their knees (eccentric phase) for >3 s. To ensure consistent movement velocity, an electronic metronome was used to guide each phase. Each experimental task was repeated thrice with a 3-s resting period between repetitions. All the experimental tasks were performed under the supervision of the physiotherapist to ensure adherence to the standardized time allocation and hip joint angle. Particularly, the pelvis rotated slightly dorsally in the ER position and ventrally in the IR position; however, all participants confirmed and practiced the experimental technique with a physiotherapist before the experiment to ensure minimal pelvic movement during hip abduction. The hip joint position for each task was maintained from the start to the end of the experiment. Raw waveforms were monitored during the experimental tasks. Therefore, if noise occurred during the test, we marked it as a failed attempt and retested.

### Data analysis

The EMG amplitude was separately expressed as the root mean square (RMS) for each muscle for each phase—early concentric, late concentric, hold, early eccentric, and late eccentric. For each phase, the RMS was calculated for 1 s from the mean of the samples over time. The RMS of each phase was normalized to the peak 1 s RMS obtained during 3 s MVIC test and calculated as the percentage MVIC (%MVIC). The %MVIC was used to calculate the RAR of the gluteal muscle-to-TFL (Lee et al., 2014). Participants were excluded if non-interpretable noise was identified.

No participants were entirely excluded from the analysis (*n* = 11). However, specific trials were excluded if uninterpretable noise was identified. The number of excluded data points per muscle and position was as follows: GMin-a, flexion and IR (FIR) position (*n* = 2), other positions (*n* = 1); GMin-p, flexion and ER position (*n* = 3), neutral (N), ER, FIR, extension and neutral (EN), and extension and external rotation (EER) positions (*n* = 2), other positions (*n* = 1); PIRI, FIR and EN positions (*n* = 2), other positions (*n* = 1); GMax, neutral, flexion and neutral, and FIR (*n* = 1), GMed; FIR position (*n* = 1), TFL; FIR position (*n* = 1), RF; FIR and extension and IR positions (*n* = 1). Consequently, the final sample size for each task ranged from 8 to 11 participants.

### Statistical analysis

All statistical analyses were performed using IBM SPSS Statistics for Mac, Version 29.0 (Armonk, NY, USA: IBM Corp, Released 2020). Two-way ANOVA and *post-hoc* tests (Bonferroni’s test) were used to compare the EMG amplitudes and RAR of each muscle between the three rotational joint positions (neutral, IR, and ER) and between the five phases for each sagittal joint position (neutral, flexion, and extension). Additionally, two-way ANOVA were used to compare EMG amplitudes between GMin-a and GMin-p segments across phases, with *post-hoc* tests performed only when significant interactions were detected. Statistical significance was set to 0.05.

## Results

### EMG amplitude of the hip muscles between hip joint positions and phases

**Figure 3:**
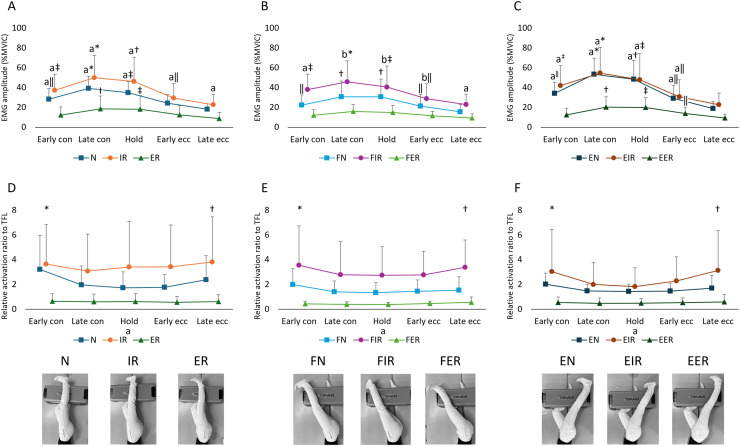
EMG amplitudes of the GMin-p and RAR for GMin-p to TFL across hip positions and phases. (A and D) neutral position, (B and E) flexion position, (C and F) extension position. ^a, b^ indicate a significant difference in each hip joint position. ^*, †, ‡, ∥^ indicate a significant difference in each phase. A significant interaction was observed between (A) and (C). (A) ^a^ *vs*. neutral and external rotation positions; *P* < 0.05; (B) ^a^ *vs*. other positions; *P* < 0.05, ^b^ *vs*. flexion and external rotation positions; *P* < 0.05; (C) ^a^ *vs*. extension and external rotation positions; *P* < 0.05. (A)–(C) * *vs*. other phases; *P* < 0.05, ^†^ *vs*. early concentric, early eccentric, and late eccentric; *P* < 0.05, ^‡^ *vs*. early eccentric and late eccentric; *P* < 0.05, ^∥^ *vs*. late eccentric; *P* < 0.05. A significant effect was observed for (D)–(F). (D) ^a^ *vs*. neutral and external rotation position; *P* < 0.05, * *vs*. late concentric, hold, and early eccentric; *P* < 0.05, ^†^ *vs*. early eccentric; *P* < 0.05. (E) and (F) ^a^ *vs*. other phases; *P* < 0.05, * *vs*. late concentric and hold; *P* < 0.05, ^†^ *vs*. early eccentric; *P* < 0.05. N, neutral position; IR, neutral and internal rotation positions; ER, neutral and external rotation positions; FN, flexion and neutral rotation positions; FIR, flexion and internal rotation positions; FER, flexion and external rotation positions; EN, extension and neutral positions; EIR, extension and internal rotation positions; EER, extension and external rotation positions, con, concentric; ecc, eccentric; TFL, tensor fascia latae; GMin-p, posterior segment of gluteus minimus; EMG, electrography; RAR, relative activation ratio.

**Figure 4:**
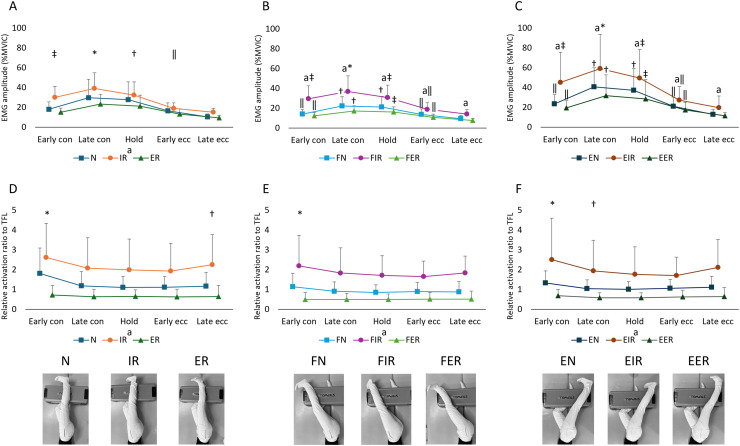
EMG amplitudes of the GMed and RAR for GMed to TFL across hip positions and phases. (A and D) Neutral position, (B and E) flexion position, (C and F) extension position. ^a^ indicates a Significant difference in each hip joint position. ^*, †, ‡, ∥^ indicate a significant difference in each phase. A significant interaction was observed between (B) and (C). (A) ^a^ *vs*. neutral and external rotation position; *P* < 0.05, * *vs*. other phases; *P* < 0.05, ^†^ *vs*. early concentric, early eccentric, and late eccentric; *P* < 0.05, ^‡^ *vs*. early eccentric and late eccentric; *P* < 0.05, ^∥^ *vs*. late eccentric; *P* < 0.05. (B) and (C) ^a^ *vs*. other positions; *P* < 0.05, * *vs*. other phases; *P* < 0.05, ^†^ *vs*. early concentric, early eccentric, and late eccentric; *P* < 0.05, ^‡^ *vs*. early eccentric and late eccentric; *P* < 0.05, ^∥^ *vs*. late eccentric; *P* < 0.05. A significant effect was observed for (D)–(F). (D)–(F) ^a^ *vs*. other positions; *P* < 0.05. (D) * *vs*. other phases; *P* < 0.05, ^†^ *vs*. early concentric; *P* < 0.05. (E) * *vs*. late concentric, hold, and early eccentric; *P* < 0.05. (F) * *vs*. late concentric, hold, and early eccentric; *P* < 0.05, ^†^ *vs*. hold; *P* < 0.05. N, neutral position; IR, neutral and internal rotation positions; ER, neutral and external rotation positions; FN, flexion and neutral rotation positions; FIR, flexion and internal rotation positions; FER, flexion and external rotation positions; EN, extension and neutral positions; EIR, extension and internal rotation positions; EER, extension and external rotation positions; con, concentric; ecc, eccentric; TFL, tensor fascia latae; GMed, gluteus medius; EMG, electromyography; RAR, reactive activation ratio.

**Figure 5:**
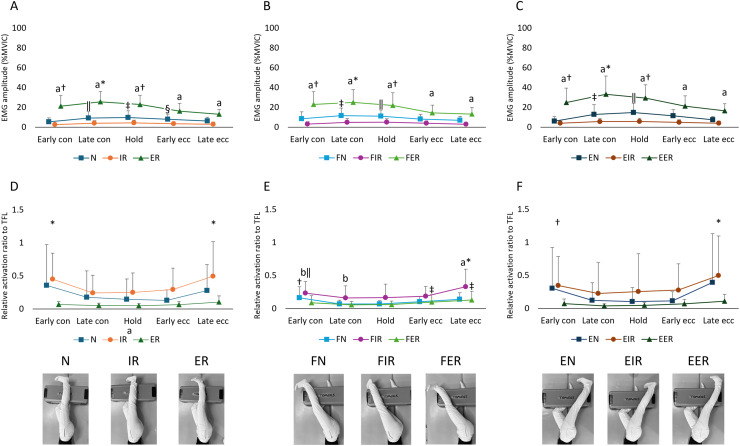
EMG amplitudes of the RF and RAR for RF to TFL across hip positions and phases. (A and D) Neutral position, (B and E) flexion position, (C and F) extension position. ^a^ indicates a Significant difference in each hip joint position. ^*, †, ‡, ∥, §^ indicate a significant difference in each phase. There were significant interactions between (A)–(C) and (E). (A)–(C) ^a^ *vs*. other positions; *P* < 0.05, (A) * *vs*. other phases; *P* < 0.05, ^†^ *vs*. early eccentric and late eccentric; *P* < 0.05, ^‡^ *vs*. early concentric and late eccentric; *P* < 0.05, ^∥^ *vs*. early eccentric; *P* < 0.05, ^§^ *vs*. late eccentric; *P* < 0.05. (B) and (C) * *vs*. hold, early eccentric, and late eccentric; *P* < 0.05, ^†^ *vs*. early eccentric and late eccentric; *P* < 0.05, ^‡^ *vs*. early concentric and late eccentric; *P* < 0.05, ^∥^ *vs*. late eccentric; *P* < 0.05. (E) ^a^ *vs*. other positions; *P* < 0.05, ^b^ *vs*. flexion and external rotation positions; *P* < 0.05, * *vs*. other phases; *P* < 0.05, ^†^ *vs*. late concentric, hold, and early eccentric; *P* < 0.05, ^‡^ *vs*. late concentric and hold; *P* < 0.05, ^∥^ *vs*. late concentric; *P* < 0.05. A significant main effect was observed in (D) and (F). (D) ^a^ *vs*. neutral and internal rotation positions; P < 0.05, * *vs*. late concentric, hold, and early eccentric positions, *P* < 0.05. (F) * *vs*. other phases; *P* < 0.05, ^†^ *vs*. late concentric and hold; *P* < 0.05. N, neutral position; IR, neutral and internal rotation positions; ER, neutral and external rotation positions; FN, flexion and neutral rotation positions; FIR, flexion and internal rotation positions; FER, flexion and external rotation positions; EN, extension and neutral positions; EIR, extension and internal rotation positions; EER, extension and external rotation positions; con, concentric; ecc, eccentric; TFL, tensor fascia latae; RF, rectus femoris; EMG, electromyography; RAR, relative activation ratio.

**Figure 6:**
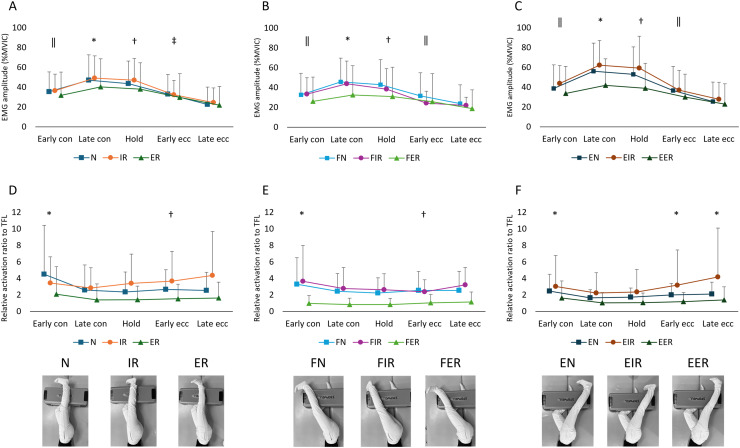
EMG amplitudes of the GMin-a and RAR for GMin-a to TFL across hip positions and phases. (A and D) Neutral position, (B and E) flexion position, (C and F) extension position. No significant interactions were observed between (A) and (E). ^*, †, ‡, ∥^ indicate a significant main effect in each phase. (A)–(C) * *vs*. other phases; *P* < 0.05, ^†^ *vs*. early concentric, early eccentric, and late eccentric; *P* < 0.05, ^‡^ *vs*. early concentric and late eccentric; *P* < 0.05, ^∥^ *vs*. late eccentric; *P* < 0.05. (D) and (E) * *vs*. late concentric and hold; *P* < 0.05, ^†^ *vs*. hold; *P* < 0.05. (E) * *vs*. late concentric concentration and hold; *P* < 0.05. N, neutral position; IR, neutral and internal rotation positions; ER, neutral and external rotation positions; FN, flexion and neutral rotation positions; FIR, flexion and internal rotation positions; FER, flexion and external rotation positions; EN, extension and neutral positions; EIR, extension and internal rotation positions; EER, extension and external rotation positions; con, concentric; ecc, eccentric; TFL, tensor fascia latae; GMin-a, anterior segment of gluteus minimus, EMG, electromyography; RAR, relative activation ratio.

**Figure 7:**
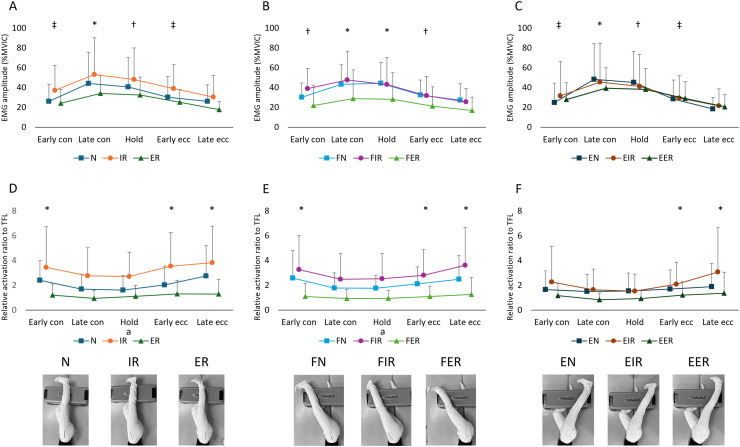
EMG amplitudes of the PIRI and RAR for PIRI to TFL across hip positions and phases. (A and D) Neutral position, (B and E) flexion position, (C and F) extension position. No significant interaction was observed between (A) and (E). ^a^ indicates a significant main effect in each hip joint position. ^*, †, ‡^ indicate a significant main effect in each phase. (A) and (C) * *vs*. other phases; *P* < 0.05, ^†^ *vs*. early concentric, early eccentric, and late eccentric; *P* < 0.05, ^‡^ *vs*. late eccentric; *P* < 0.05. (B) * *vs*. early concentric, early eccentric, and late eccentric; *P* < 0.05, ^†^ *vs*. late eccentric; *P* < 0.05. (D) ^a^ *vs*. neutral and external rotation positions; *P* < 0.05. (E) ^a^ *vs*. flexion and external rotation positions; *P* < 0.05. (D)–(E) * *vs*. late concentric concentration and hold; *P* < 0.05. N, neutral position; IR, neutral and internal rotation positions; ER, neutral and external rotation positions; FN, flexion and neutral rotation positions; FIR, flexion and internal rotation positions; FER, flexion and external rotation positions; EN, extension and neutral positions; EIR, extension and internal rotation positions; EER, extension and external rotation positions; con, concentric; ecc, eccentric; TFL, tensor fascia latae; PIRI, piriformis; EMG, electromyography; RAR, relative activation ratio.

**Figure 8:**
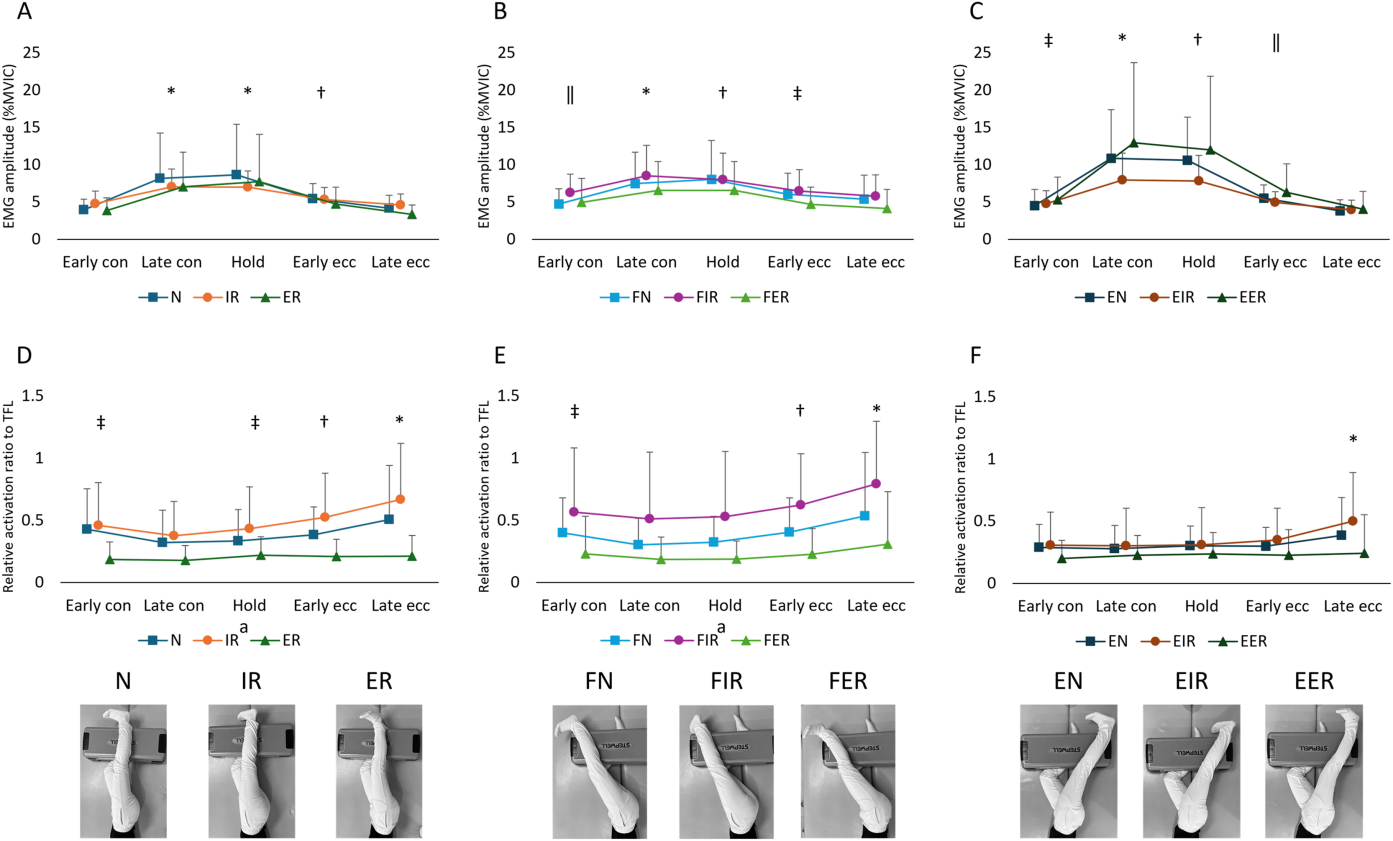
EMG amplitudes of the GMax and RAR for GMax to TFL across hip positions and phases. (A and D) Neutral position, (B and E) flexion position, (C and F) extension position. No significant interaction was observed between (A) and (E). ^a^ indicates a significant main effect of each hip joint position. ^*, †, ‡, ∥^ indicate a significant main effect in each phase. (A) * *vs*. early concentric, early eccentric, and late eccentric; *P* < 0.05, ^†^ *vs*. late eccentric; *P* < 0.05. (B) * *vs*. other phases; *P* < 0.05, ^†^ *vs*. early concentric, early eccentric, and late eccentric; *P* < 0.05, ^‡^ *vs*. early concentric and late eccentric; *P* < 0.05, ^∥^ *vs*. late eccentric; *P* < 0.05. (C) * *vs*. other phases; *P* < 0.05, ^†^ *vs*. early concentric, early eccentric, and late eccentric; *P* < 0.05, ^‡^ *vs*. early eccentric and late eccentric; *P* < 0.05, ^∥^ *vs*. late eccentric; *P* < 0.05. (D) ^a^ *vs*. external rotation position; *P* < 0.05, * *vs*. late concentric, hold, and early eccentric; *P* < 0.05, ^†^ *vs*. late concentric and hold; *P* < 0.05, ^‡^ *vs*. late concentric; *P* < 0.05. (E) ^a^ *vs*. flexion and external rotation position; *P* < 0.05, * *vs*. other phases; *P* < 0.05, ^†^ *vs*. late concentric and hold; *P* < 0.05, ^‡^ *vs*. late concentric; *P* < 0.05. (F) * *vs*. early concentric, late concentric, and early eccentric; *P* < 0.05. N, neutral position; IR, neutral and internal rotation positions; ER, neutral and external rotation positions; FN, flexion and neutral rotation positions; FIR, flexion and internal rotation positions; FER, flexion and external rotation positions; EN, extension and neutral positions; EIR, extension and internal rotation positions; EER, extension and external rotation positions; con, concentric; ecc, eccentric; TFL, tensor fascia latae; GMax, gluteus maximus ; EMG, electromyography; RAR, relative activation ratio.

**Figure 9:**
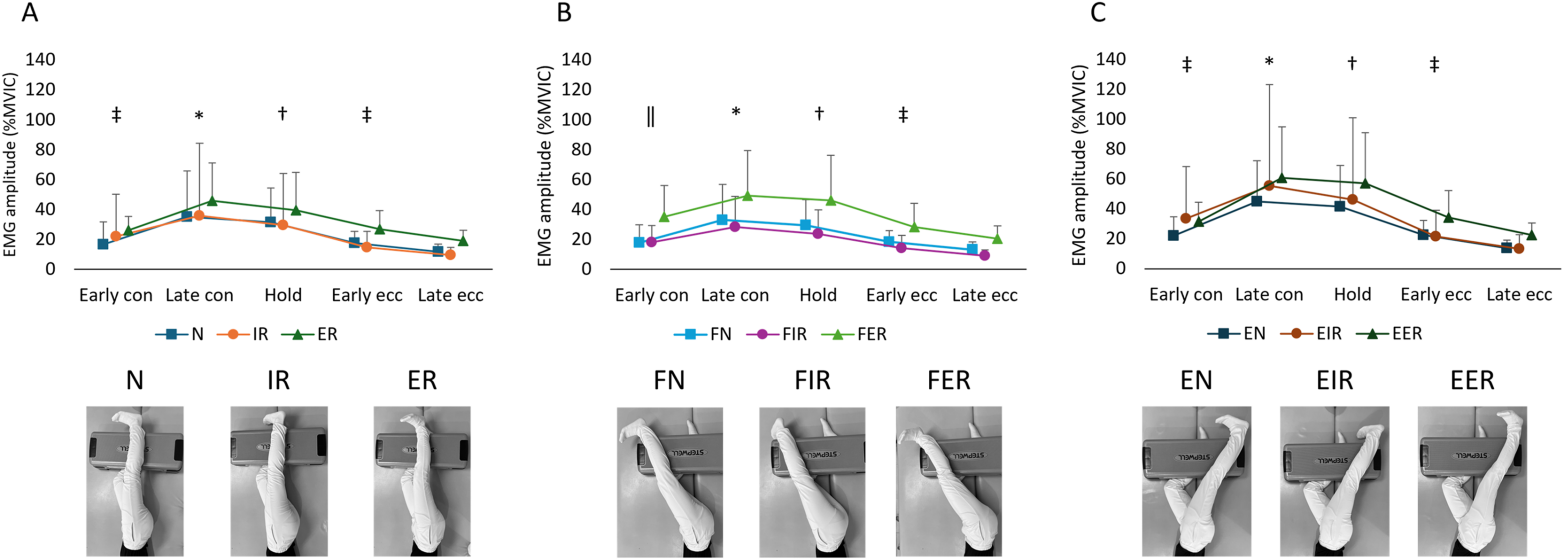
EMG amplitudes of the tensor fascia latae in neutral position (A), flexion position (B), and extension position (C) during each phase. No significant interaction was observed between (A) and (C). ^*, †, ‡, ∥^ indicate a significant main effect in each phase. (A) and (C) * *vs*. other phases; *P* < 0.05, ^†^ *vs*. early concentric, early eccentric, and late eccentric; *P* < 0.05, ^‡^ *vs*. late eccentric; *P* < 0.05. (B) * *vs*. other phases; *P* < 0.05, ^†^ *vs*. early concentric, early eccentric, and late eccentric; *P* < 0.05, ^‡^ *vs*. early eccentric and late eccentric; *P* < 0.05, ^∥^ *vs*. late eccentric; *P* < 0.05. N, Neutral position; IR, neutral and internal rotation positions; ER, neutral and external rotation positions; FN, flexion and neutral rotation positions; FIR, flexion and internal rotation positions; FER, flexion and external rotation positions; EN, extension and neutral positions; EIR, extension and internal rotation positions; EER, extension and external rotation positions; con, concentric; ecc, eccentric; EMG, electromyography.

We observed significant interactions between the hip joint position and phase in the GMin-p, GMed, and RF groups (Figs. 3–5). Focusing on the EMG amplitude phase, the GMin-p, GMed, and RF was significantly higher during the late concentric phase. No significant interaction was observed between GMin-a, PIRI, GMax, and TFL; however, a significant main effect of the phase was observed (the highest during the late concentric phase). Regarding the hip joint position of the EMG amplitude, the GMin-p and GMed were significantly higher in the extension and IR (EIR) positions, and RF was significantly higher in the EER positions. No significant main effect of the hip joint position was observed in GMin-a, PIRI, GMax, or TFL (Figs. 6A‒6C, 7A‒7C, 8A‒8C, and 9).

The GMin-p EMG amplitude during the late concentric phase was significantly higher in the N and IR positions (N, 39.3 ± 12.2 %MVIC; *P* = 0.00, IR, 50.0 ± 22.6 %MVIC; *P* = 0.00, Fig. 3A) than in the neutral and ER position, in the FIR position (FIR, 45.8 ± 21.4 %MVIC; *P* = 0.00, Fig. 3B) than in the flexion and ER (FER) position, in the EN and EIR (EN, 53.5 ± 16.3 %MVIC; *P* = 0.00, EIR, 54.7 ± 26.0 %MVIC; *P* = 0.00, Fig. 3C) than in the EER position.

**Figure 10:**
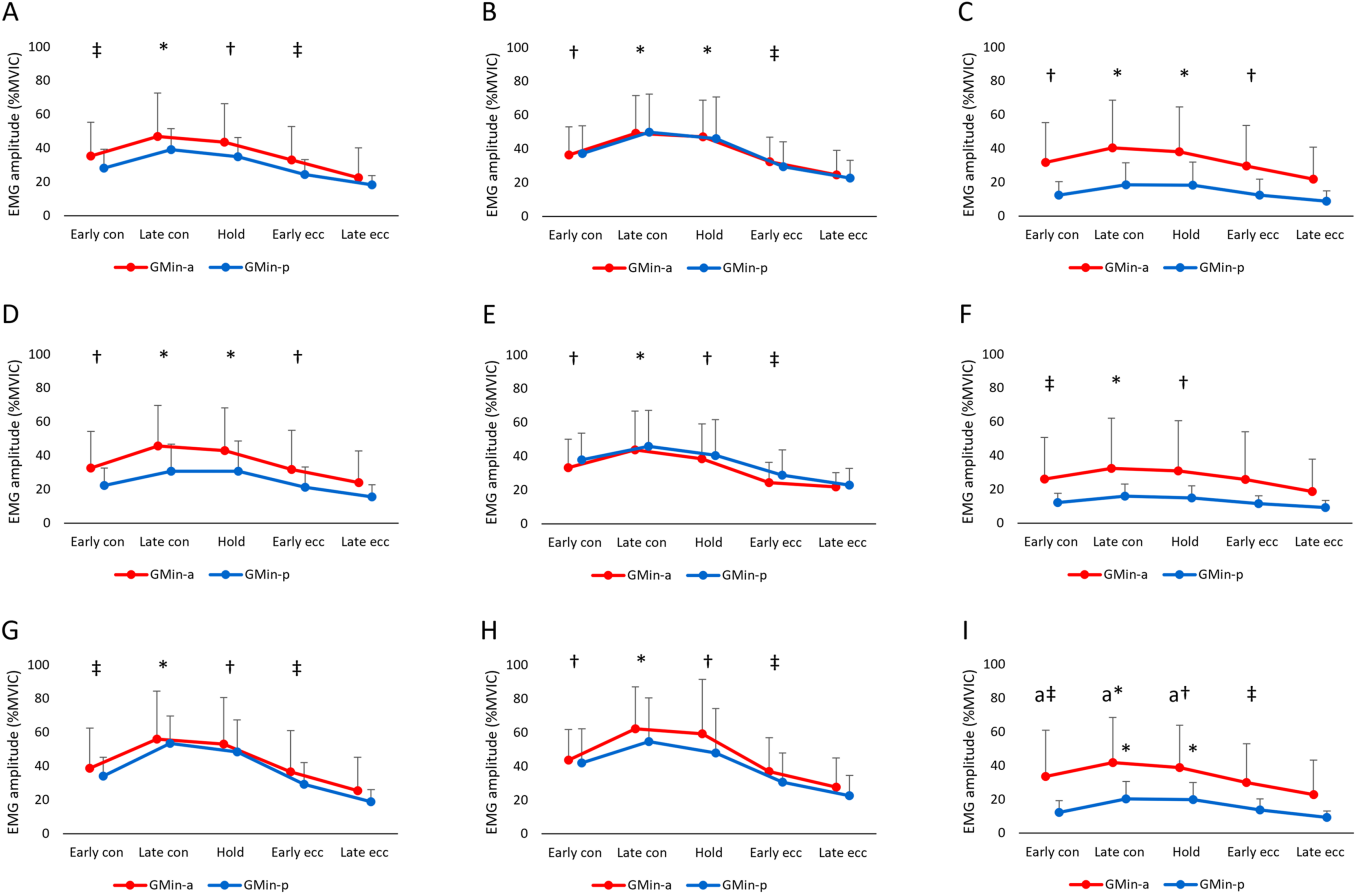
Comparison of EMG amplitudes between anterior and posterior segments of gluteus minimus across nine hip positions. (A) Neutral position, (B) internal rotation position, (C) external rotation position, (D) flexion position, (E) flexion and internal rotation position, (F) flexion and external rotation position, (G) extension position, (H) extension and internal rotation position, (I) extension and external rotation position. ^a^ indicates a significant difference in each hip joint position. ^*, †, ‡^ indicate a significant difference in each phase. There was significantly interaction in (I). (A) and (G) * *vs*. other phases; *P* < 0.05, ^†^ *vs*. early concentric, early eccentric, and late eccentric; *P* < 0.05, ^‡^ *vs*. late eccentric; *P* < 0.05. (B) and (F) * *vs*. early concentric, early eccentric, and late eccentric; *P* < 0.05, ^†^ *vs*. early eccentric, and late eccentric; *P* < 0.05, ^‡^ *vs*. late eccentric; *P* < 0.05. (C) and (D) * *vs*. early concentric, early eccentric, and late eccentric; *P* < 0.05, ^†^ *vs*. late eccentric; *P* < 0.05. (E) and (H) * *vs*. other phases; *P* < 0.05, ^†^ *vs*. early eccentric, and late eccentric; *P* < 0.05, ^‡^ *vs*. late eccentric; *P* < 0.05. (I) ^a^ *vs*. posterior segment of gluteus minimus, * *vs*. early concentric, early eccentric, and late eccentric; *P* < 0.05, ^†^ *vs* early eccentric, and late eccentric; *P* < 0.05, ^‡^ *vs*. late eccentric; *P* < 0.05. GMin-a, anterior segment of gluteus minimus; GMin-p, posterior segment of gluteus minimus; con, concentric; ecc, eccentric; EMG, electromyography.

Regarding the comparison between GMin segments, a significant interaction between the segment and phase was observed only in the EER position. In the EER position, the EMG amplitude of the GMin-a was significantly higher than that of the GMin-p. In contrast, no such segment-specific differences were observed in other hip positions (Fig. 10).

The GMed EMG amplitude during the late concentric phase was significantly higher in the FIR position (FIR: 36.8 ± 15.8 %MVIC, *P* = 0.00, Fig. 4B), and subsequently in other positions, EIR position (EIR: 59.2 ± 34.6 %MVIC, *P* = 0.00, Fig. 4C) than in the other positions. The RF EMG amplitude during the late concentric phase in all sagittal positions was significantly higher in the ER and EER positions than in the other positions (Figs. 5A‒5C).

### RAR of the hip muscle-to-TFL

A significant main effect was observed for GMin-p, PIRI, GMax, GMed, and RF in the hip joint position and for all muscles in the phase. For the RARs of GMin-p to the TFL, only the IR position showed a significant difference compared with the ER position (Fig. 3D), whereas the FIR and EIR positions were higher than all other positions (Figs. 3E, 3F). For the RARs of the GMed to the TFL, IR, FIR, and EIR positions were significantly higher than those in all positions (Figs. 4D‒4F). For the RARs of PIRI and GMax to the TFL, the IR and FIR positions were significantly higher than the ER and FER positions (Figs. 7D, 7E; 8D, 8E). GMin-a, GMin-p, PIRI, GMax, and GMed were significantly higher in the early concentric and/or late eccentric phases in all hip positions. For the RARs of the RF to the TFL, the FIR position and late eccentric phase were significantly higher than those in the other positions and phases, whereas they were <1 (Fig. 5E).

## Discussion

This study investigated the muscle activities of the hip abductors—including the GMin and PIRI—and determined the RAR for each hip muscle-to-TFL during abduction in different hip positions.

### EMG amplitude of the hip muscles

Our study found that the hip position that activated both GMin-p and GMed involved hip IR. Notably, the EMG amplitudes for the GMin-p and GMed were highest in the EIR position. To interpret the effects of the hip position, it is important to consider that the moment arms and the line of action of the muscles involved in hip abduction change according to the hip position (Dostal, Soderberg & Andrews, 1986; Delp et al., 1999). In the hip IR position, the entire lower limb is in an IR position, and the insertion of the GMin-p and GMed are displaced anteroinferiorly. According to the musculoskeletal model by Delp et al. (1999), altering the hip joint position significantly changes the moment arm of the hip abductors. Specifically, the IR position may alter the muscle’s line of action, potentially enhancing the mechanical advantage of the GMin-p and GMed for hip abduction in a gravity-resisting direction (Dostal, Soderberg & Andrews, 1986). Furthermore, the GMin-p and GMed have the potential for hip extension (Neumann, 2010), which may be emphasized in the hip EIR position.

In addition, side-lying hip abduction in all external rotation positions had the highest RF activation. This result is also influenced by the fact that the anatomical position of the RF relative to gravity changes according to the hip position. According to the mechanical analysis by Dostal, Soderberg & Andrews (1986), hip external rotation is changed to the line of action of the muscles and changing the number of the muscles involved in hip abduction. In the hip external rotation position, the attachment site of the RF faced upward. Placing the muscle in a more direct antigravity position during hip abduction and increased mechanical contribution of the hip abduction. In contrast, the insertion of the RF was displaced toward the floor (in a no-gravity position) in the hip IR position, which decreased its mechanical contribution to the hip abduction movement.

Additionally, segmental differences within the GMin were observed in the EER position. However, a comparison of GMin-a (Fig. 6) and GMin-p (Fig. 3) across all positions suggests that GMin-a activity was relatively unaffected by hip position, whereas GMin-p activity was specifically minimized in the EER position. In the EER position, the GMin-p is placed in a maximally shortened state (Beck et al., 2000), Furthermore, the increased activity of superficial hip muscles, such as RF (Fig. 5) and TFL (Fig. 9), likely creates a compensatory environment that further inhibits GMin-p activation. In contrast, both segments co-activated in all other positions—most notably in those involving hip IR. These results suggest that the EER position leads to the selective inhibition of the GMin-p due to superficial hip muscle compensation, position involving hip IR position allows the GMin to be activated as a single functional unit.

**Figure 11:**
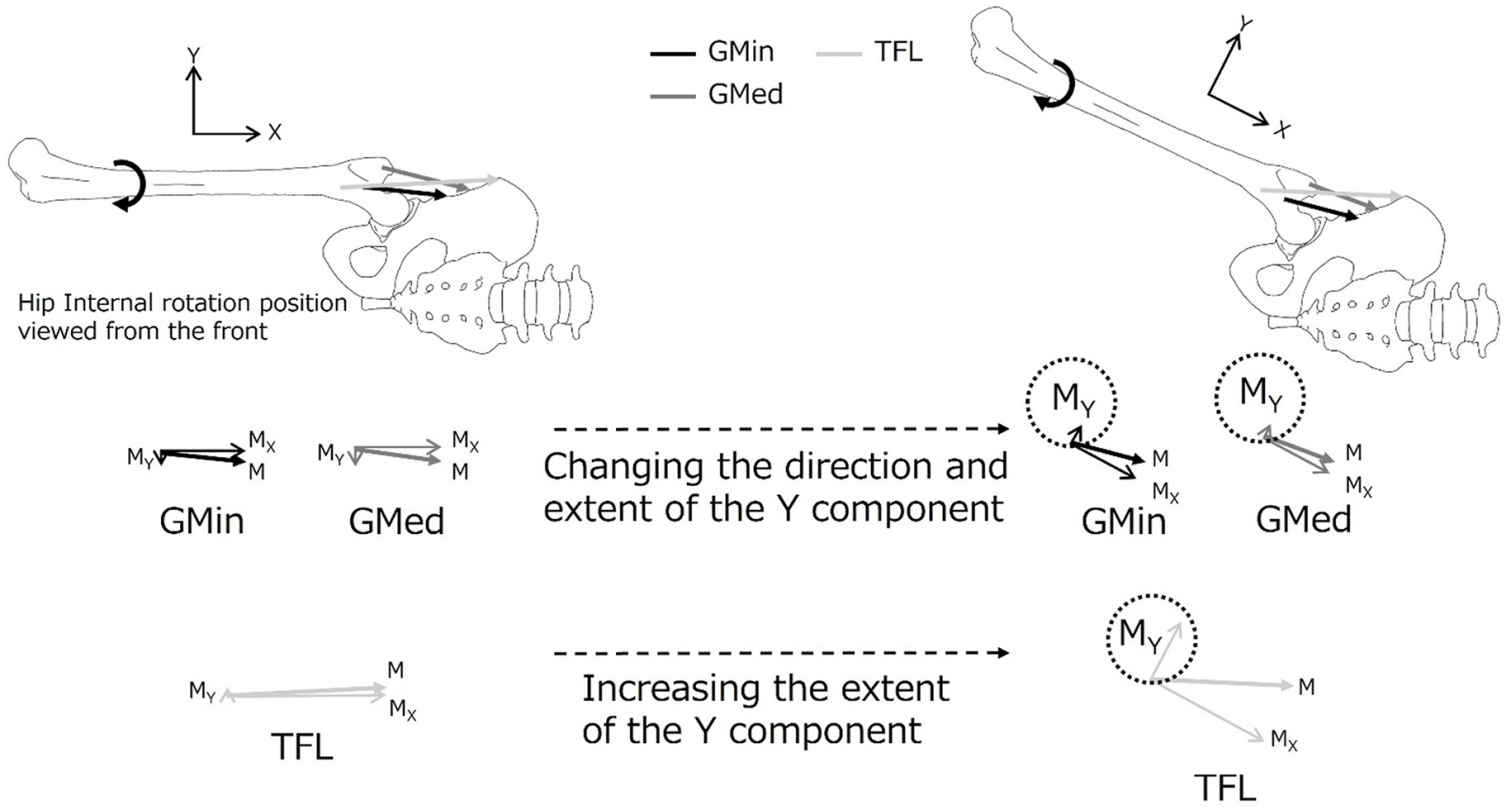
Change in the resolution of the force vectors of the GMin, GMed, and TFL, with changes in hip position. GMin, gluteus minimus; GMed, gluteus medius; TFL, tensor fascia latae.

Moreover, the EMG amplitudes of the GMin-p and GMed were significantly higher in the late concentric phase, whereas those of the hip abductors tended to be high in the same phase. Therefore, considering the influence of the force vector of each muscle during each phase was necessary. In Fig. 11, the force vectors of the GMin-p and GMed are resolved into the X and Y components, respectively, during each abduction phase. The Y-components in the hip IR position were changed to increase the internal joint moment of the hip abduction in the late concentric phase. Consequently, these muscles contributed to the abduction movements in the late concentric phase. Previous studies comparing the phases of GMin activity during hip abduction showed that GMin-p activity was significantly greater than GMin-a activity in the hip IR position (Semciw et al., 2014). Furthermore, in a study comparing the activities of the hip abductors during side-lying hip abduction in different hip joint positions, the EMG amplitude of the GMed was significantly higher in the IR position (Lee et al., 2014). The increased in EMG amplitude during the late concentric phase, despite the muscles reaching a more shortened position, suggests a neurophysiological compensation strategy. According to the length-tension relationship, the force-generating capacity of a muscle typically decreases as it shortens. Increased the recruitment of motor units to counteract this mechanical disadvantage and maintain the required abduction torque at the late concentric phase. Therefore, the observed activity reflects a selective recruitment motor strategy aimed at optimizing joint stability and torque production at the late concentric phase. In addition, an unexpected result was that muscle activity was highest during the late concentric phase rather than during the hold phase because the hip abduction angle was set at 30° in this study, and the experimental task was not performed to the end range of motion. Although the hip joint movements are different, the muscle activity of the psoas major was maximized during straight leg raising held at the end range of motion (Okubo et al., 2021). As a joint approaches the end range of motion, the muscles must be activated at a shorter length, which increases motor unit recruitment to stabilize the joint and compensate for the decreased force-generating capacity (length-tension relationship). In our study, because the 30° hold position was not the end of range, the muscles could maintain enough length to produce torque relatively efficiently, requiring less compensatory motor unit recruitment than during the late concentric phase or a hold at the end-range of motion. Therefore, maintaining hip abduction at the end range of motion might maximize the muscle activity of the hip abductor muscles. Our new finding in this study was the increase in the EMG amplitudes of GMin-p and GMed in the hip EIR position and during the late concentric phase.

### RAR of the hip muscle-to-TFL

The position where the hip muscle-to-TFL RAR was significantly higher differed by muscle: the GMin-p and GMed were all in IR positions; PIRI and GMax were in the IR and FIR positions, and the RF was in the IR position. The most important phases of these RARs were the early concentric and late eccentric phases. This finding is similar to that of a previous study that found a higher GMed-to-TFL ratio during side-lying hip abduction in the hip IR position (Lee et al., 2014). As shown in Fig. 5, the Y component of the TFL was extremely small during the early concentric or late eccentric phase but became larger as the hip abduction angle increased. Thus, EMG values in these phases were lower, and the activity ratios of GMin, PIRI, GMax, and GMed predominated that of the TFL, as the TFL activity was minimized during the early concentric or late eccentric phase. Consequently, side-lying hip abduction in the IR or FIR position selectively activate all major hip abductors, including GMin-p, PIRI, and GMed. The RAR of the GMax and RF to the TFL was <1 but significantly increased in the hip IR or FIR positions. This finding is consistent with that of a previous study reporting that TFL activity exceeds that of superior GMax during side-lying hip abduction (Selkowitz, Beneck & Powers, 2013). TFL activity was greater than that of GMax and RF during side-lying hip abduction. Therefore, our findings indicate that the side-lying hip abduction movement in the hip IR or FIR position minimized TFL activity during the early concentric or late eccentric phase and selectively activated GMin-p, PIRI, and GMed.

### Study implications

Our study findings have important implications for side-lying hip abduction exercises. Selective activation exercise is considered important for GMin and proper execution of side-lying hip abduction exercises, depending on the purpose. The results of this study demonstrated that changing the joint position at the start of the side-lying hip abduction exercise and the degree of hip abduction may activate different muscles. Notably, empty cans (shoulder internal rotation and slight abduction) or full can exercises used in shoulder joint rehabilitation activate the supraspinatus, infraspinatus, and subscapularis (Escamilla et al., 2009). Furthermore, shallow elevation minimizes upper trapezius activity (Timmons et al., 2015). These reports suggest that shoulder empty can exercises, which control rotation and abduction angles, effectively optimize rotator cuff activation while minimizing trapezius activity. While acknowledging the anatomical and functional differences between the shoulder and hip joints, our findings suggest a potential functional analogy. If side-lying hip abduction exercises were performed involving hip IR positions and slight hip abduction movement, the activities of the GMin-p, GMed, and PIRI would be maximized, and TFL activity would be minimized. Therefore, we propose that this specific movement pattern could be conceptualized as a ‘hip empty can’ exercise. Furthermore, side-lying hip abduction exercise in the EIR position is recommended as the hip joint position that best activates the GMin-p and GMed. Specifically, the activity levels of the GMin-p and GMed were 54 %MVIC and 59 %MVIC, respectively. The activity levels are 40–60 %MVIC (high) required for muscle strengthening (Macadam & Feser, 2019), suggesting that side-lying hip abduction in the hip extension and IR positions may be a useful exercise for strengthening these muscles.

### Limitation

This study has some limitations. First, obtaining high-quality data from several joint positioning tasks is challenging owing to the invasive nature of the intramuscular wire electrodes used to record the GMin and PIRI EMGs. Thus, excluding data from the three participants for some tasks was necessary to ensure that high-quality data were included in the analysis. A previous study reported challenges encountered by studies using intramuscular wire electrodes (Okubo et al., 2021). In addition, surface EMG was used for the GMax, GMed, and TFL. Second, as this study involved the use of invasive fine-wire electrodes for the GMin and PIRI, careful consideration was required regarding their invasiveness when selecting other muscles for recording. Previous studies have addressed the issue of crosstalk by using wire electrodes to measure GMax, GMed, and TFL activity (Selkowitz, Beneck & Powers, 2013, 2022, 2023). In addition, using surface electrodes for GMax raises concerns, as the signals might be attenuated by the adipose tissue. Our study findings must be interpreted with consideration of these tissues. Furthermore, this study used a common electrode placement for the GMax, whereas previous studies demonstrated differences in the activation of the upper and lower GMax during side-lying hip abduction and gait (Lyons et al., 1983; Selkowitz, Beneck & Powers, 2016). Therefore, future research is needed to determine whether the muscle activity of each fiber of the GMax changes depending on the hip joint position during side-lying hip abduction movement. We were unable to quantify the hip joint position and compensatory movements of the other joints during each trial. Moreover, the hip rotation position was defined as the maximum ER or IR, and no angle was specified. If the participants were unable to maintain the specified hip position or if compensatory movement of other joints was confirmed, they had to repeat the task; thus, the results of this study were not significantly affected. However, in experimental tasks, hip flexion or extension combined with rotation is often unable to control the slight inclination of the pelvis. Given that it did not affect the raw data during experimental tasks, it was unavoidably included as a successful task. Therefore, the quantification of the hip joint position and pelvis during each trial may have further supported our findings. Finally, this study focused exclusively on side-lying hip abduction, making it difficult to directly compare the results with other functional tasks such as lunges, step-ups, or squats. While our findings provide detailed insights into the influence of hip rotation and elevation phases during hip abduction, future research should examine these variables in the context of other therapeutic exercises to provide more comprehensive clinical recommendations for gluteal exercise.

## Conclusions

Our study compared the activity patterns of the hip abductor muscles during side-lying hip abduction movements in different hip joint positions. The side-lying hip abduction movement activated the gluteal muscles in the internal hip rotation position and showed different activation patterns in the early and late concentric phases. Side-lying hip abduction during the late concentric phase with EIR position caused maximal hip abductor activity. Conversely, side-lying hip abduction during the early concentric phase involving the IR position activates the gluteal muscles while minimizing TFL activation.

## Supplemental Information

10.7717/peerj.21536/supp-1 Supplemental Information 1 Raw data of EMG amplitudes and RAR for hip muscles to TFL.

## Additional Information and Declarations

### Data Availability

The following information was supplied regarding data availability:

The raw data is available in the Supplemental File.
